# Cellular heterogeneity of the developing worker honey bee (*Apis mellifera*) pupa: a single cell transcriptomics analysis

**DOI:** 10.1093/g3journal/jkad178

**Published:** 2023-08-07

**Authors:** Anirudh Patir, Anna Raper, Robert Fleming, Beth E P Henderson, Lee Murphy, Neil C Henderson, Emily L Clark, Tom C Freeman, Mark W Barnett

**Affiliations:** The Roslin Institute, University of Edinburgh, Easter Bush, Midlothian EH25 9RG, UK; The Roslin Institute, University of Edinburgh, Easter Bush, Midlothian EH25 9RG, UK; The Roslin Institute, University of Edinburgh, Easter Bush, Midlothian EH25 9RG, UK; The Queen's Medical Research Institute, Centre for Inflammation Research, University of Edinburgh, Edinburgh BioQuarter, Edinburgh EH16 4TJ, UK; Edinburgh Clinical Research Facility, Western General Hospital, University of Edinburgh, Edinburgh EH4 2XU, UK; The Queen's Medical Research Institute, Centre for Inflammation Research, University of Edinburgh, Edinburgh BioQuarter, Edinburgh EH16 4TJ, UK; Institute of Genetics and Cancer, Western General Hospital, University of Edinburgh, Edinburgh EH4 2XU, UK; The Roslin Institute, University of Edinburgh, Easter Bush, Midlothian EH25 9RG, UK; The Roslin Institute, University of Edinburgh, Easter Bush, Midlothian EH25 9RG, UK; The Roslin Institute, University of Edinburgh, Easter Bush, Midlothian EH25 9RG, UK; Beebytes Analytics CIC, The Roslin Innovation Centre, University of Edinburgh, The Charnock Bradley Building, Easter Bush, Midlothian EH25 9RG, UK

**Keywords:** honey bee, *Apis mellifera*, single-cell RNA-Seq, network analysis, metamorphosis, development

## Abstract

It is estimated that animals pollinate 87.5% of flowering plants worldwide and that managed honey bees (*Apis mellifera*) account for 30–50% of this ecosystem service to agriculture. In addition to their important role as pollinators, honey bees are well-established insect models for studying learning and memory, behavior, caste differentiation, epigenetic mechanisms, olfactory biology, sex determination, and eusociality. Despite their importance to agriculture, knowledge of honey bee biology lags behind many other livestock species. In this study, we have used scRNA-Seq to map cell types to different developmental stages of the worker honey bee (prepupa at day 11 and pupa at day 15) and sought to determine their gene expression signatures. To identify cell-type populations, we examined the cell-to-cell network based on the similarity of the single-cells transcriptomic profiles. Grouping similar cells together we identified 63 different cell clusters of which 17 clusters were identifiable at both stages. To determine genes associated with specific cell populations or with a particular biological process involved in honey bee development, we used gene coexpression analysis. We combined this analysis with literature mining, the honey bee protein atlas, and gene ontology analysis to determine cell cluster identity. Of the cell clusters identified, 17 were related to the nervous system and sensory organs, 7 to the fat body, 19 to the cuticle, 5 to muscle, 4 to compound eye, 2 to midgut, 2 to hemocytes, and 1 to malpighian tubule/pericardial nephrocyte. To our knowledge, this is the first whole single-cell atlas of honey bees at any stage of development and demonstrates the potential for further work to investigate their biology at the cellular level.

## Teaser

In this study Patir et al. have used scRNA-Seq to map cell types to different developmental stages of the worker honey bee (prepupa at day 11 and pupa at day 15), and sought to determine their gene expression signatures. To the authors’ knowledge, this is the first whole single cell atlas of honey bees at any stage of development and demonstrates the potential for further work to investigate their biology at the cellular level.

## Introduction

The western honey bee, *Apis mellifera*, is valued for the pollination services it provides to many crops and wild flowers (Corbet 1991; Klein et al. 2007; Gallai et al. 2009; Breeze et al. 2011; Ollerton et al. 2011; Kleijn et al. 2015) as well as for its production of honey and wax (Hepburn et al. 1991; Carreck 2018). Globally there are 11 species of honey bee (Engel 1999; Arias and Sheppard 2005) whose distribution is restricted to Asia with the exception of the western honey bee found all over the world and indigenous to Africa, the Middle East, and Europe (Seeley 1985; Ruttner 1988). Despite the diversity of honey bee species in Asia, the world's beekeeping industry is based almost entirely on one species, *A. mellifera*. In addition to their importance to agriculture and the economy, honey bees represent a useful model organism for many areas of research (Elekonich and Roberts 2005; Dearden et al. 2009). Although Hymenoptera and Diptera diverged over 300 million years ago (Misof et al. 2014), honey bees are similar in terms of their physiology and other characteristics to the best-studied model organism in the phylum Arthropoda, *Drosophila melanogaster*.

The genome for the western honey bee was first published in 2006 by the Honey Bee Genome Sequencing Consortium (Honeybee Genome Sequencing Consortium 2006). This was later improved upon by Elsik et al. (2014) who found c.5,000 more protein-coding genes, 50% more than previously reported. Wallberg et al. (2019) reported a further improvement using Pac-Bio long reads (Amel_HAv3.1). Parallel to annotating the genome, efforts have also been made to associate phenotypes with genes using omic analyses. Studies have examined changes in gene expression associated with different treatments (pheromones and pesticide) and how they relate to behavior, phenotype, and changes associated with the colony e.g. queen loss (Christen et al. 2016; Chaimanee and Pettis 2019; Ma et al. 2019). Pheromone and pesticide treatment effects on gene expression have also been studied in combination with various conditions, e.g. with seasonal changes (Jeon et al. 2020), infections from Varroa (Navajas et al. 2008; Zhang et al. 2010; Morfin et al. 2019) and Nosema (Li et al. 2016; Badaoui et al. 2017; Azzouz-Olden et al. 2018). Mechanisms underlying developmental processes such as embryogenesis, ageing, and caste determination have also been analyzed from an omics perspective (Evans and Wheeler 1999; Tsuchimoto et al. 2004; Azevedo et al. 2011; Yin et al. 2018; He et al. 2019). Whilst some of the aforementioned experiments have derived transcriptomic data from whole honey bees, others have studied tissue-specific differences e.g. analysis of differences in alternate splicing patterns between the brain and fat body (Wang et al. 2012; Zayed and Robinson 2012; Kannan et al. 2019). However, a comprehensive tissue/cell atlas of the developing honey bee is still lacking.

Bulk tissue transcriptomics atlases have been used effectively to annotate and assign function to poorly annotated genes in pig, sheep, mice, humans, and *D. melanogaster* (Su et al. 2002; Chintapalli et al. 2007; Freeman et al. 2012; Clark et al. 2017; Leader et al. 2018). scRNA-Seq enables the classification of cell subtypes which is challenging with solely a bulk RNA-Seq strategy. Single-cell expression atlases have been derived from several tissues for the *Tabula Muris* which spans 100,000 cells across 20 mouse tissues (Tabula Muris Consortium 2018). Other efforts like the Fly Cell Atlas have conducted exhaustive scRNA-Seq studies on individual tissues providing a comprehensive atlas, e.g. for the brain (Davie et al. 2018) and midgut (Hung et al. 2020) of *D. melanogaster*. Studies have also tracked the development of various organisms including *Drosophila* (Karaiskos et al. 2017), zebrafish (Raj et al. 2018), cnidarians (Sebé-Pedrós, Saudemont et al. 2018)) and *Caenorhabditis elegans* (Packer et al. 2019). Such studies have demonstrated the sensitivity of scRNA-Seq data in tracking cell types, their cell-specific developmental lineages and providing an estimate of how conserved gene expression signatures are across species.

The aim of this study was to generate single-cell transcriptomics data for two stages of worker honey bee development. To achieve this aim, we have generated scRNA-Seq data from a prepupal stage (day 11) and a pupal stage (day 15). These two stages were selected to capture cellular diversity immediately before and after the rearrangement of the larval to adult body plan. In holometabolous insects, the larvae and adults have very different body plans enabling them to exploit different resources. Although the larvae of social insects and solitary bees have subsequently evolved to be relatively immobile, this remarkable evolutionary development, facilitating resource partitioning across developmental stages, contributed to holometabolous insects comprising over half of global eukaryotic diversity (Belles 2017). Despite the importance of metamorphosis in the evolutionary success of insects, the mechanisms governing it are not completely understood. In this study, we develop approaches through from single-cell isolation to the analysis of the resultant scRNA-Seq data using gene coexpression networks (GCN) to demonstrate that generating a gene expression atlas of the whole honey bee at the level of single cells is possible at prepupal and pupal stages. At each developmental stage, we aim to identify several potential cell types and their associated gene expression signatures to better understand fundamental biology of the honey bee at a cellular level during these key stages of development.

## Material and methods

### Whole *A. mellifera* pupae cell dissociation and sorting

**Fig. 1.**
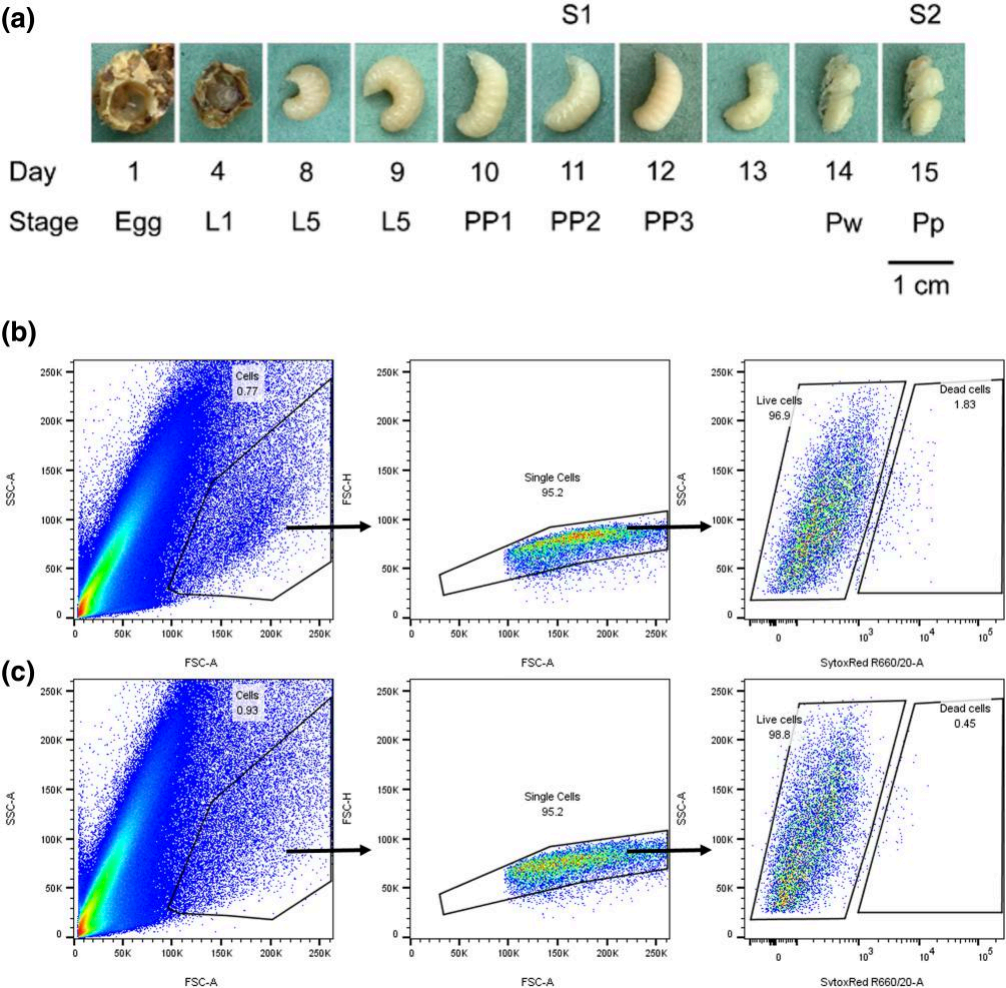
Worker honey bee development and FACS. a) Development of honey bee worker from egg to day 15 pupa. Queen bee was trapped on a broodless drawn broodframe in a queen excluder cage for 1 day and removed, samples of eggs, larvae, and pupae were taken at 1 day intervals from frame within excluder cage after queen removal. L1, 1st larval instar; L5, 5th larval instar; PP1, prepupal phase 1; PP2, prepupal phase 2; PP3, prepupal phase 3; Pw, white-eyed pupa; Pp, pink-eyed pupa. S1 and S2 were stages analyzed for single-cell transcriptomics. Representative gating strategy for live single-cell sort of stage 1 b) and stage 2 c) bee pupae. The cells gate was defined on size and granularity, and then single cells were defined using forward scatter area verses height. Live cells were then sorted by discriminating SytoxRed positive cells.

Honey bees are holometabolous and worker prepupae at day 11 (S1) and pupae at day 15 (S2) were chosen for this study in order to capture the key developmental stages between capping of the larval cell (day 9) and the emergence of the imago on day 21 (Oertel 1930 ) (Fig. 1a). To gather samples, a piece of brood comb containing appropriately staged pupae was collected from a single honey bee colony at the Easter Bush Campus apiary in August 2018. Pupae were removed from the comb and placed in microcentrifuge tubes on ice. Each pupa was placed in 0.5 ml HyQTase (GE Healthcare, Chicago, IL, USA), finely chopped with small spring scissors for 1 min, and incubated for 5 min at 25°C. Samples of each stage were centrifuged at 400 RCF for 5 min at 4°C. Cell pellets were resuspended in 1 ml WH2 medium by drawing liquid into and out of pipette tip 15 times (Goblirsch et al. 2013). Samples (*n* = 4 per stage) were pooled (total volume 4 ml), and the cells passed into a 5 ml tube through a 70 µm strainer cap (Becton, Dickinson and Company, Franklin Lakes, NJ, USA) to remove debris and aggregated cells. Following centrifugation of the filtered cells at 400 RCF for 5 min at 4°C, the supernatant was discarded, and the cells resuspended in 2 ml WH2 medium. After further centrifugation at 400 RCF for 5 min at 4°C, cells were resuspended in 1 ml WH2 medium and stained with 1:2,000 Sytox Red (Thermo Fisher, Waltham, MA, USA) for downstream cell viability analysis during cell sorting. Gating strategies sorted cells on the basis of their size (forwards vs side scatter area to exclude debris), single cells (forward scatter area vs height to exclude doublet cells), and viability using a 633 nm laser and 660/20 band pass emission filter on an Aria IIIu FACS (Becton, Dickinson and Company, NJ, USA) (Fig. 1b and c). Before sequencing, the cells were counted and tested again for viability using a TC20 automated cell counter (Bio-Rad, Hercules, CA, USA).

### Single-cell RNA-Seq data generation, processing, and quality control

Approximately 7,000 cells at each stage were used for cDNA library preparation using the Chromium platform v2.0 (10X Genomics, Pleasanton, CA, USA), as per the manufacturer's instructions. Library quality was confirmed with a LabChip Gx24 bioanalyzer (PerkinElmer, Waltham, MA, USA). Sequencing (75 bp paired-end) was performed using an Illumina NextSeq550 platform using a Mid Output 150 cycle flow cell (Clinical Research Facility, University of Edinburgh).

Binary base call files were preprocessed using the Cell Ranger pipeline (10X Genomics 2023). Reads were assigned to sample index tags to generate FASTQ files. Of the total 180 million reads generated, 69 million were mapped to sample indices of prepupa (day 11) and 55 million to pupa (day 15). For read alignment, the recent *A. mellifera* reference genome (Amel_HAv3.1) and annotation (GFF file) were downloaded from NCBI. To keep compatibility with Cell Ranger, the GFF file was converted to GTF using the Cufflinks software suite (Tuxedo) (Trapnell et al. 2012), and only protein-coding regions were considered.

The resultant GTF file and reference genome were used to generate an expression matrix for each sample. Raw expression matrices were quality controlled and analyzed using the Seurat package v2 in R, using the default thresholds (Stuart et al. 2019). Data from the two developmental stages were merged then cells with a low number of UMI reads ≤700 and ≥10% being mitochondrial were filtered out. Furthermore, genes expressed in ≤ 3 cells were removed (Stuart et al. 2019). The data were log-normalized, and genes with the most variable expression across cells were identified, i.e. possessing a standard deviation >0.5 and an average expression between 0.0125 and 3. Effects from technical factors, including variable library sizes and percent mitochondrial UMIs, were regressed out. The scaled variables were reduced to a lower feature space using principal component (PC) analysis. The most significant PCs (61 in total, *P* value < 0.05) based on JackStraw permutations (Chung and Storey 2015) were considered and the resultant cell vs PC matrix was loaded into the network analysis tool, Graphia (Freeman et al. 2022). A correlation (Pearson similarity coefficient) matrix was then calculated between cells comparing the PC profile of each cell. Using this cell similarity matrix, a cell-to-cell network was constructed where cells (represented by a node) were connected to the 20 most similar cells by an edge, while only considering similarities beyond a Pearson cut-off threshold *r* ≥ 0.77. This graph was clustered using Markov clustering algorithm (Van Dongen 2008) with an inflation value of 1.6. Cells were further filtered to remove those with an edge degree lower than three. For statistical purposes, small clusters with less than 10 cells were merged into the closest cluster with the highest sum of weighted edges.

### Gene coexpression network analysis

Gene expression modules associated with biological process and cell types were identified using gene coexpression network (GCN) analysis. For conventional transcriptomics, data GCNs are widely used to capture coexpressed clusters of genes associated with a shared biological function (Nirmal et al. 2018; Patir et al. 2019; Patir et al. 2020). However, due to the inherent variability within scRNA-Seq data attributed to the transcriptional heterogeneity of cells and the technical effects of dropouts (false zero expression values) (Hicks et al. 2018), we were unable to capture these coexpressing genes as they are poorly correlated. Hence, we have averaged expression values across cells within a cluster to improve the stability of signals within clusters whilst also highlighting intercell type variation rather than the variation within a cell type (Satija and Shalek 2014). Such cell-aggregation approaches have been widely used to improve the signal-to-noise ratio in scRNA-Seq [e.g. (Baran et al. 2019; Persad et al. 2023)].

Through cluster analysis i.e. grouping of similar data, we aimed to identify which cells cluster together based on the similarity of their gene expression profiles, thus revealing biologically relevant groups of cells or “cell clusters” potentially representing cell types or cell states. Translating this to genes, we identify those which share a similar expression profile or coexpress across cells or in this study, aggregates of similar cells. These groups of genes or “cluster of genes” potentially represent a particular biological function or pathway. Before averaging reads, filters were applied to reduce the effects of technical artifacts and low-level signals, these are described as follows. First, for a given cluster of genes, cells were assigned a zero expression value if: (1) fewer than three cells within the cluster expressed that gene, (2) the maximum expression across cells was < 0.5 logged transcript per million (TPM), and (3) < 5% of cells within the clusters expressed that gene. Moreover, to avoid the influence of outliers or spikes in expression commonly observed in RNA-Seq data, we capped the maximum expression of a gene to the 95% percentile from cells of the cluster. The gene expression from the resultant filtered data was then averaged across cells for each cluster. Where a cluster consisted of cells derived from both developmental stages, they were averaged separately for each stage. In this way, the 63 cell clusters identified from the graph analysis of cells, were expanded to 81 stage differentiated cell clusters. Consequently, an expression matrix of genes vs cell clusters was used to generate a GCN within Graphia. Only genes with a maximum expression above 0.2 average logged TPM were considered. The k-nearest neighbor algorithm was applied where each cell was connected to the four most similar cells provided this similarity was *r* ≥ 0.7. Subsequently, the graph was clustered using the Louvain cluster algorithm (Blondel et al. 2008) applied with a granularity setting of 0.65. Differential gene coexpression analysis was performed using the default Wilcox test provided in Seurat to gauge the magnitude and specificity of genes towards cell clusters and developmental stages based on their expression profiles.

### Functional gene annotation using *D. melanogaster* homologs

Functional annotation of clusters of genes from the GCN analysis was provided based on gene ontology (GO) enrichment analysis and literature mining. We followed a similar procedure to (Sebé-Pedrós, Saudemont, et al. 2018 and Sebé-Pedrós, Chomsky, et al. 2018). First, each protein of the bee proteome was mapped to the most similar (E score < 10^−4^) protein in *D. melanogaster* (Release 6 plus ISO1 MT) based on their sequence using BLASTp (Altschul et al. 1990). The resultant nomenclature in combination with studied honey bee genes was used to functionally annotate clusters of genes. Furthermore, the *Drosophila* homologs were also used for GO enrichment analysis, this was conducted for each cluster of genes using the clusterProfiler package in R (Yu et al. 2012) with the genome-wide annotation for *Drosophila* (org.Dm.eg.db) as the reference GO term database (Carlson et al. 2016). For literature mining, previous publications and resources were used including the *Drosophila* FlyAtlas2 (Leader et al. 2018) and Honey Bee Protein Atlas (Chan et al. 2013).

## Results

### The expanding cellular diversity of the developing pupa

For this study we developed a cell isolation protocol for the pre-pupal and pupal stages (S1, prepupa at day 11; S2, pupa at day 15) (Fig. 1a) of the honey bee, which provided sufficient cell numbers and viability for processing through the 10x Chromium platform v2.0. Four prepupae or pupae samples were combined for each stage. These cells were then sorted based on their size, granularity, and staining to identify viable single cells (Fig. 1b and c). Just before library preparation, the cells went through a second round of counting and viability testing to assure sufficient cells were processed for sequencing.

Raw reads from the scRNA-Seq experiment were mapped to the NCBI-based *A. mellifera* (Amel_HAv3.1) genome using the Cell Ranger pipeline from 10X Genomics. Sixty-nine million reads mapped to samples from the day 11 S1 sample and 55 million reads to the day 15 S2 sample. After filtering and removal of outlier samples, 9,119 genes for developmental stage S1 and 9,309 genes for developmental stage S2 were identified (Table 1). In comparison, there are 9,944 protein-coding genes in the honey bee genome Amel_HAv3.1.

**Table 1.** Number of genes at each step of the analysis for stages 1 and 2 samples.

	Analysis = steps | data type (cells/pseudobulk)			
	1) Raw data | Cells	2) Quality control | Cells	3) Cell clustering & aggregation | Pseudobulk	4) Gene clustering | Pseudobulk
Stage 1	9,477	9,119	9,119	3,994
Stage 2	9,586	9,309	9,309	3,994

Cells were filtered on their read content, removing cells with a low read count (<700 per cell) and those with a high mitochondrial gene content (>10%), leaving 2,148 cells from S1 and 2,178 cells from S2 (Table 2).

**Table 2.** Number of cells or pseudobulk samples at each step of the analysis for stages 1 and 2 samples.

	Analysis = steps | data type (cells/pseudobulk)		
	(1) Raw data | Cells	(2) Quality control | Cells	(3) Cell clustering and aggregation | Pseudobulk
Stage 1	2,444	2,148	30
Stage 2	2,365	2,178	51

**Fig. 2.**
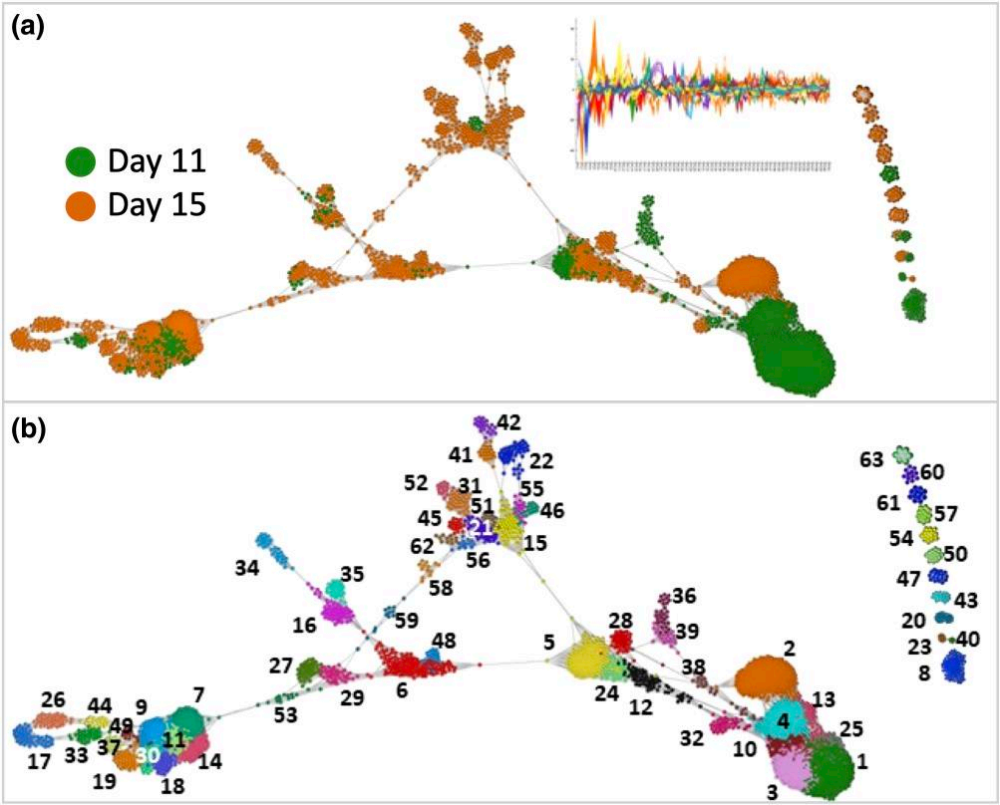
Honey bee cell populations as defined by scRNA-Seq analysis. a) Cell-to-cell network generated by comparing the 61 most significant PCs for each cell. See insert in a) showing plot of PCA profiles (y axis, each PC signified by color) for all cells (x axis) in the graph. Each of the node represents an individual cell and the edges the 10 most significant correlations between them *r* threshold > 0.77. The graph is composed of 4,149 cells connected by 31,000 edges. In a), nodes are colored by the pupal stage from which they were derived. Note the clustering of some cells based on stage, suggesting stage-specific cell populations. In b), nodes are colored according to their cluster ID, 63 clusters being defined. The clusters disconnected from the central network are positioned on the right. Numbers indicate cluster ID.

As the two samples were from a single batch, datasets were merged and followed the standard scRNA-Seq preprocessing steps of normalization and scaling (for mitochondrial content and library size). To cluster cells based on their gene expression profile, the 1,361 most variable genes were identified and were reduced using PC analysis from which the 61 most significant PCs were inspected. These PCs were used to calculate Pearson pairwise similarity between cells across the merged dataset thereby generating a cell-to-cell similarity matrix. The matrix was used to construct a cell-to-cell network (Fig. 2) where each node represented a cell and those having a Pearson correlation coefficient greater than *r* ≥ 0.77 were connected to one another by an edge. Furthermore, for each cell, only the 20 nearest neighbors were considered and poorly connected cells, i.e. connected to <3 other cells, were removed. These steps further helped in removing potential outlier cells that were dissimilar to the majority of cells. The final cell-to-cell graph consisted of 4,149 nodes (cells) (2,045 cells from S1 and 2,104 cells from S2) and 31,000 edges.

The cell-to-cell graph consisted of one large, interconnected component and 11 smaller components. Cells from the two stages were distributed differently across the network indicative of stage-specific cell types with S2 possessing more heterogenous populations of cell types (Fig. 2a). On studying the distribution of genes and reads across cells, cells from S2 showed a significant (1.28 times, *P* value < 10^−3^) increase in the number of genes expressed relative to S1. Clustering of the cell network resulted in 72 clusters potentially representing distinct cell types of states. To improve the statistical power of downstream analyses, smaller cell clusters with less than 10 cells were merged with a neighboring cluster to which they were highly connected, i.e. had the highest sum total of weighted (based on the Pearson correlation) connections resulting in 63 cell clusters (Table 3 and Fig. 2b). Interestingly, even though the number of cells from both stages was approximately the same, 50 clusters comprised of cells from S2, while S1 cells were only present in 30 clusters. The exact distribution of stages across cell clusters is shown in Supplementary Fig. 1. All together, these results were indicative of the expanding cellular diversity in the developing honey bee pupa.

**Table 3.** Annotation of the 63 cell clusters represented in fig. 2b.

Tissue/cell type	S1	S2	S1 and S2	Number of Unique Cell Clusters
Neuron	C11, C54	C7, C30, C37	C9, C14, C18, C19, C49	10
Sense organ	—	C29, C53	C27	3
Eye	—	C17, C26, C44	C33	4
Glia	—	C61	C16, C35	3
Fat body	C1, C3, C4, C10, C13, C25	C2	—	7
Hemocyte	—	—	C20, C43	2
Midgut	C8	C50	—	2
Malpighian tubule or pericardial nephrocyte	—	C63	—	1
Muscle	—	C28	C5, C12, C24, C32	5
Cuticle	C36, C39, C51	C15, C21, C31, C41, C42, C45, C46, C52, C55, C56, C58, C59, C62, C22, C47	C38	19
Unknown	C23	C34, C40, C48, C57, C60	C6	7
Total number of cell clusters				63

### Clustering of coexpressing genes and their functional annotation

**Fig. 3.**
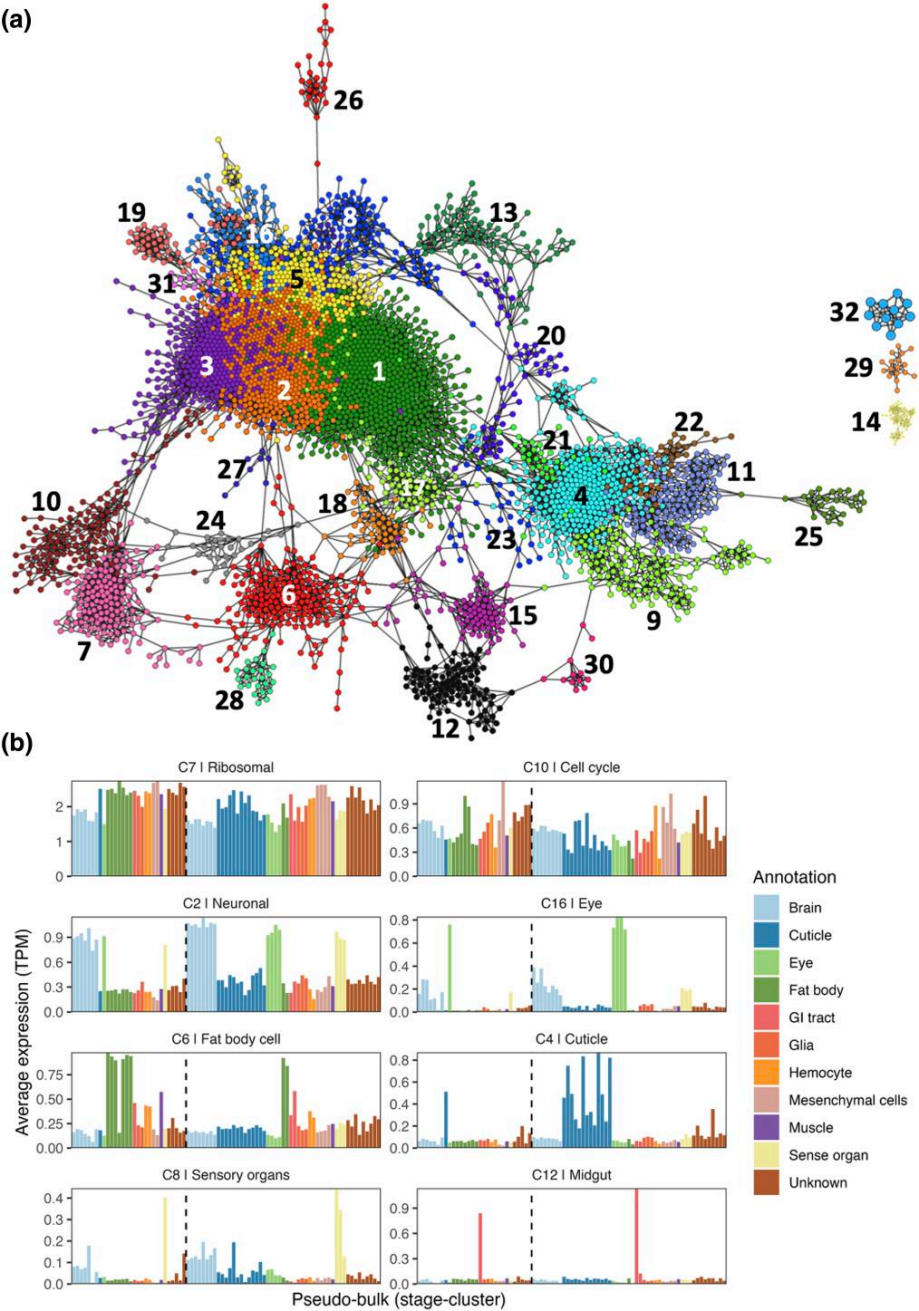
Gene correlation network analysis of expression profile of genes across cell clusters. a) GCN composed of 3,994 nodes (genes) connected by 11,400 edges where *r* threshold > 0.7. Nodes are colored according to Louvain cluster (granularity 0.65). b) Average expression profile of clusters of genes based on each gene's average expression across a cluster of cells. To the left of the dotted line are cell clusters from the day 11 prepupa and on the right of the line are cell clusters from the day 15 pupa. Clusters of cells have been grouped based on similarity.

**Fig. 4.**
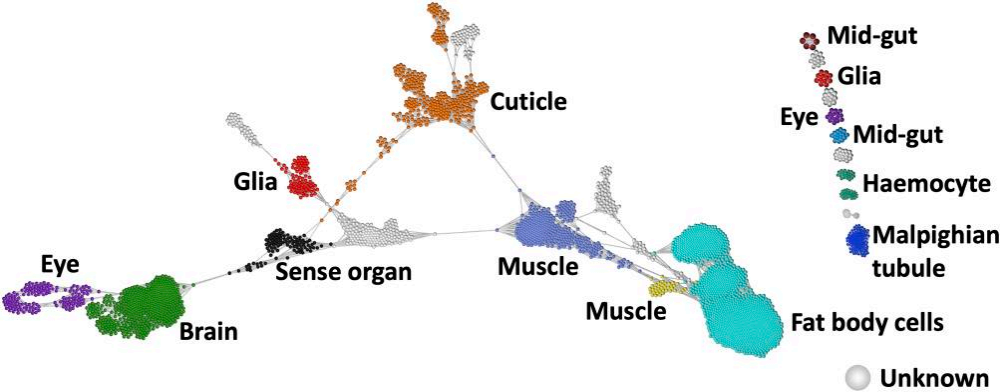
Final assignment of cell identity. The cell-to-cell network is similar to that from Fig. 3 where each dot represents a cell with similar cells connected to one another. However, it is overlayed with broad level annotation (color) for the various cell clusters that have been defined based on GCN analysis, Fly Atlas2, Honey Bee Protein Atlas, and literature mining. Clusters where we could not find sufficient supporting evidence are classed as “Unknown” in gray.

A stage-cluster vs gene expression matrix was used to calculate a gene-to-gene correlation matrix, from which we constructed a GCN. In the network, genes were connected to the four most similar genes by an edge provided they were highly correlated *r* ≥ 0.7. The network graph consisted of 3,994 genes which were clustered into 32 clusters of genes using the Louvain clustering algorithm with a granularity of 0.65 (Fig. 3 and Supplementary Table 1). The expression profile of each of the 32 clusters of genes is shown in Supplementary Fig. 2. Tissues, cell types, and biological processes corresponding to the clusters of genes in the GCN were identified from GO enrichment (Supplementary Table 1), public resources, and literature mining (Supplementary Table 2), the final annotation of which is summarized in Figs. 3 and 4 and Table 4.

**Table 4.** Identity, gene coexpression cluster ID, stage of expression, and gene count for the 32 clusters shown in fig. 3.

Gene Coexpression Cluster ID	Identity	Stage of Expression and Cell Cluster	Gene Count
1	Gene with higher expression in S2 cells	S1 and S2	708
2	Neuron	S1 and S2	541
3	Neuron	S1 and S2	359
4	Cuticle	Majority S2	315
5	Neuron	S1 and S2	252
6	Fat body	Majority S1	199
7	Ribosomal proteins	S1 and S2	145
8	Sensory organs	S1 and S2	136
9	Cuticle	S1 and S2	136
10	Cell cycle	S1 and S2	125
11	Cuticle	S2	114
12	Midgut	S2—cell cluster 50	95
13	Glia	S1 and S2	94
14	Muscle	S1 and S2	88
15	Unknown	S1	81
16	Compound eye	S1 and S2	80
17	Unknown	S2—Cell cluster 57	76
18	Malpighian tubule or pericardial nephrocyte	S2—Cell cluster 63	60
19	Neurons and sensory organs, eye	S2	54
20	Sensory organs	S1 and S2	53
21	Unknown	S2—cell cluster 47	37
22	Cuticle	S2	32
23	Higher expression in non-neural tissues at S2	S1 and S2	31
24	Proteosome	S1 and S2	29
25	Unknown	S1—cell cluster 23 S2—cell cluster 40	27
26	Cuticle	S2—cell clusters 41 and 42	26
27	Sensory organs	S1 and S2	25
28	Hemocyte	S1 and S2	25
29	Unknown	S2—cell cluster 60	17
30	Unknown	S2—cell cluster 22	12
31	Electron transport chain	S1 and S2	11
32	Hemocyte	S1 and S2	11

The enrichment analysis was performed on each cluster of genes based on the *D. melanogaster* GO reference database. For this analysis, honey bee genes based on their corresponding proteins were first mapped to the *Drosophila melanogaster* proteome using blastp (Altschul et al. 1997) where the most similar mapping was considered for a gene. Twenty-six clusters were found to be enriched in various GO terms (adj. *P* value < 0.05) (Supplementary Table 1).

These analyses revealed clusters of genes associated with stage-specific differences in expression profiles, as well as tissue/cell-specific biology, e.g. neuronal, muscle, cuticle, fat body, alimentary canal, and hemolymph:

#### Stage-specific clusters

The largest cluster of genes, cluster 1 comprised of 708 genes (Fig. 3) with a higher expression in cells from S2 relative to S1. GO terms enriched in these genes included those related to development, the top three GO terms being “postembryonic animal morphogenesis” (adj. *P* value = 2.29 × 10^−13^), “instar larval or pupal morphogenesis” (adj. *P* value = 3.35 × 10^−13^) and “regulation of intracellular signal transduction” (adj. *P* value = 5.39 × 10^−13^). Some of the other clusters of genes that showed stage-associated expression were clusters 4, 6, 11, 12, 15, 16, 18, 19, 21, 22, 23, 25, 26, 29, and 30, in total comprising 1,884 genes.

#### Neuronal-related cell clusters

Three clusters (2, 3, and 5) contained genes associated with various neuronal biology and were highly expressed in 17 cell clusters identified as being related to neurons and sense organs. All of the cell clusters identified as neuronal or sense organ-related expressed both synapsin (for some of the related cell stage-cluster groups Log_2_ fold change > 0.26, adj. *P* value < 1.28 × 10^−3^) and the nicotinic acetylcholine receptor alpha 1 subunit (*nAChRa2*, Log_2_ fold change > 0.57, adj. *P* value < 8.9 × 10^−4^), while those annotated as only neuronal expressed the NR1 subunit of the NMDA receptor. Some genes were expressed differentially across developmental stages, including genes from the family of G protein-coupled receptors that bind octopamine and/or tyramine. Octopamine is widely distributed in the nervous system of invertebrates where it acts as a neurotransmitter (Verlinden et al. 2010) and is thought to be the functional homolog of vertebrate adrenergic transmitters. On examining the different classes of these G protein-coupled receptors (Sinakevitch et al. 2017) in invertebrates, OA1 receptor showed a high expression in cell clusters 37 and 60 containing S2 cells (*Oa1*, Log_2_ fold change > 0.25, adj. *P* value < 2.7 × 10^−2^), *AmTAR1* was highly expressed in cell cluster 33 having cells from both pupal stages, whilst *AmTARII* showed a high expression in cell clusters 9 and 11 from S1.

#### Glial-related cell clusters

Glial cell*s* have an essential role in the development of neurons and are involved in regulation of synaptic plasticity, provide trophic support to neurons, and contribute to the blood–brain barrier (Shah et al. 2018). In the honey bee, these cells can be labeled using a serum raised against the *Drosophila* glial transcription factor *repo* (Shah et al. 2018), *repo* was highly expressed (Log_2_ fold change > 1.12, adj. *P* value < 4.2 × 10^−11^) in several non-neuronal cell clusters (6, 16, 34, 35, 48 and 61) identifying them as potentially representing glia or glial-related cells. Cluster 13 was found to be associated with these cell clusters. Further subclassification of these cells was revealed through genes linked with astrocytes in *Drosophila*, including *Eaat2* and GABA transporters (Gat-a and Gat-1b), which were differentially expressed (Log_2_ fold change> 1.7, adj. *P* value < 2.4 × 10^−2^ in cell cluster 35 (Freeman 2015).

#### Sensory organ and compound eye-related cell clusters

A higher average expression of genes from cluster 8 was observed in sensory organs relative to neuron-related cell clusters. GO terms enriched in genes from this cluster were associated with ciliary biology, the most significant terms being “cilium organization” (adj. *P* value = 6.48 × 10^−24^), “cilium assembly” (adj. *P* value = 8.53 × 10^−24^), and “plasma membrane bounded cell projection assembly” (adj. *P* value = 4.36 × 10^−21^). The modified primary cilium is a structure common to all peripheral sensory neurons in arthropods with the exception of photoreceptors (Keil 2012), suggesting that cell clusters 27, 29, and 53 were related to sense organs other than the compound eye and ocelli. Four cell clusters (clusters 26, 33, 44, and 49) identified as neural were associated with the compound eye. Genes from cluster 16 (80 genes) were specifically expressed in these eye-related cell clusters with genes associated with this tissue e.g. *AmPNR-like* (LOC413558, Log_2_ FC > 0.47, adj. *P* value < 6.5 × 10^−4^) shown by in situ hybridization to be expressed in the developing eyes of pupae in either the photoreceptor cells or support cells (Velarde et al. 2006). LOC408804 (1-phoshatidylinositol 4,5 bisphosphate phosphodiesterase epsilon-1) was expressed (Log_2_ FC > 3.04, adj. *P* value < 3.3 × 10^−21^) in these cell clusters and in Drosophila it's homolog (*Plc21c*) has a role in pigment dispersing factor neurons in the circadian photoresponse (Ni et al. 2017). Phosrestin 2 (*LOC551043*) was specifically expressed (Log_2_ FC > 0.27, adj. *P* value < 9 × 10^−3^) in cell clusters 17, 26, and 44, and has been associated with the visual system in honey bees where it has a role in circadian rhythms (Rodriguez-Zas et al. 2012).

#### Cuticle-related cell clusters

Gene clusters 4, 9, 11, 22, and 30 included genes expressed in 19 cell clusters associated with the cuticle. Only four of these cell clusters were associated with the S1 prepupal cuticle (cell clusters 36, 38, 39, and 51). This could indicate that cell populations from the S1 stage cuticle are less diverse than those from S2 which might consist of heterogenous populations of cells differentiating in different regions of the developing honey bee exoskeleton. The cuticle-associated clusters of genes included key enzymes in the chitin biosynthetic pathway linked to cuticle development and the moulting process e.g. LOC412215 (homolog of *Drosophila* gene *kkv*, a chitin synthase that catalyzes the conversion of UDP-*N*-acetylglucosamine to chitin), LOC552276 (homolog of *Drosophila* gene *cda5*), a chitin deacetylase that catalyzes the conversion of chitin to chitosan (a polymer of *β*-1,4-linked d-glucosamine residues) (Sobala and Adler 2016) and LOC551964 (homolog of *Drosophila* gene *mmy*, an enzyme required for glycan and chitin synthesis) (Araújo et al. 2005). Chitin (the polymer of N-acetyl glucosamine) is a key component of the honey bee inner procuticle, which together with the outer epicuticle forms the exoskeleton (Locke and Krishnan 1971) and the difference in cuticle structure in arthropods is due to the different expression of cuticular proteins (Magkrioti et al. 2004). In addition to chitin, the cuticle consists of various structural proteins some of which were present in the cuticle-related clusters of genes including LOC726451 (homolog of *Drosophila* gene *Cpr57A,* Log_2_ FC > 0.87, adj. *P* value < 4.33 × 10^−27^) and *Apd-3* (Log_2_ FC > 0.32, adj. *P* value <2.45 × 10^−43^) (Falcon et al. 2019).

#### Fat body-related cell clusters

In insects, the fat body, has a similar role to the liver and adipose tissue of mammals as it functions as a store for excess nutrients, synthesizes most of the hemolymph proteins, and is responsible for detoxification processes (Arrese and Soulages 2010). Various genes associated with the fat body were found in cluster 6 (199 genes) which had a high expression in seven cell clusters. The majority of these clusters comprised cells from the S1 stage (six clusters). The gene *ilp-2* (Log_2_ FC > 0.25, adj. *P* value <1.02 × 10^−3^) is expressed in both oenocytes and trophocytes (cell types found in the fat body) in the adult honey bee (Nilsen et al. 2011) and was expressed in all seven fat body-related cell clusters. A similar expression profile was observed for *mmp2* (Log_2_ FC > 0.55, adj. *P* value <3.32 × 10^−11^) which is involved in fat body remodeling during early metamorphosis in *Drosophila* (Bond et al. 2011) and Vitellogenin receptor which has been shown to be expressed in the fat body, ovary, and head of adult worker bees (Guidugli-Lazzarini et al. 2008).

#### Hemolymph-related cell clusters

In insects, hemocytes are derived from anterior mesoderm, form part of the immune system and comprise lamellocytes, crystal cells, plasmatocytes, and granulocytes (Richardson et al 2018). Granulocytes are the major phagocytic cells and are likely to play a role in clearing cellular debris and apoptotic cells during the breakdown of tissues during metamorphosis (Richardson et al 2018). Genes within clusters 28 and 32 included known hemocyte markers (*hml* and *lz*), and the average expression of these genes was higher in cell clusters 20 and 43. Interestingly, genes from cluster 28 showed a higher expression in stage 2 hemocytes while cluster 32 showed the opposite which a higher expression in stage 1 hemocytes. The marker *hml* (hemolectin/hemocytin) (Log_2_ FC > 1.4, adj. *P* value <7.03 × 10^−8^) is specifically expressed in hemocytes in *Drosophila* in embryos and larvae, while *lz* is required for the differentiation of crystal cells (Lebestky et al. 2003) and the absence of its expression results in the differentiation of a plasmatocyte. Whilst both gene were expressed highly in the hemocyte cell clusters, *lz* showed higher levels of expression in cell cluster 43 (Log_2_ FC > 1.05, adj. *P* value <8.77 × 10^−11^), suggesting that it represented crystal cells.

#### Muscle-related cell clusters

In *Drosophila*, somatic muscle, visceral muscle, and cardiac muscle develop from the mesoderm (Gunage et al. 2017). The largest somatic muscles in the honey bee are two pairs of indirect flight muscles (dorsumventral and anterior–posterior) in the thorax that are responsible for moving the wings up and down (Snodgrass 1910). Cell clusters 5, 12, 24, 28, and 32 were annotated as differentiating muscle cells based on expression of *twist* (Gunage et al. 2017), *mef2* (Crittenden et al. 2018), *nautilus* (Abmayr and Keller 1998)*, TpnT* (Domingo et al. 1998), *TpNI* (Herranz et al. 2005), *TpnCIIb* (Herranz et al. 2005), myosin heavy chain (*LOC409843*), and myosin light chain (*LOC409881*) (11390828). The gene *nautilus* (Log_2_ FC > 0.25, adj. *P* value < 3.40 × 10^−3^) may have an equivalent function to the vertebrate myogenic regulatory factors (*myoD* and *Myf5*) that act as master control genes in mesoderm to initiate the first steps of somatic muscle development (Abmayr and Keller 1998; Zammit 2017). Expression of *nautilus* specifically in cell clusters 12, 24, and 32 indicated that these cell clusters comprised of cells differentiating into somatic muscle. Expression of *twist* (Log_2_ FC > 0.78, adj. *P* value < 1.39 × 10^−3^) in *Drosophila* is required earlier in development in mesoderm definition for specification of all muscle types, *twist* was expressed specifically in cell clusters 5, 12, 24, 28, and 32, and was also expressed in cell cluster 60 (unknown identity). Cell clusters 5, 12, 24, 28, and 32 were also associated with differentiating muscle cells based on GO analysis of gene cluster 14 whose gene showed high expression in these cells relative to other cell clusters. The top GO terms for the gene cluster 14 included “striated muscle cell differentiation” (adj. *P* value = 2.02 × 10^−20^), “muscle structure development” (adj. *P* value = 4.12 × 10^−20^), and “muscle cell differentiation” (adj. *P* value = 8.61 × 10^−20^).

#### Alimentary canal

The tissue comprises four major compartments, the foregut, midgut, malpighian tubules, and hind gut (Snodgrass 1910). Genes from cluster 12 were highly expressed in cell clusters 8 and 50, with higher levels in stage 2. The associated gene cluster included alpha-glucosidase I and II shown to be expressed in honey bee ventriculus (Kubota et al. 2004), as well as organic anion transporting polypeptide genes *Oatp33Ea* (Log_2_ FC > 0.71, adj. *P* value < 6.33 × 10^−48^) and *Oatp58Dc* both of which are specific to the *Drosophila* midgut of larva and adult based on the FlyAtlas 2 tissue RNA-Seq database (Leader et al. 2018). Cell cluster 63 had a high expression of genes from cluster 18 thought to be related to malpighian tubules or pericardial nephrocytes, including *Cubilin* (Log_2_ FC > 2.99, adj. *P* value < 1.67 × 10^−141^) and *Amnionless* which in *Drosophila* mediate protein reabsorption in both malpighian tubules and pericardial nephrocytes (Zhang et al. 2013).

### Differential gene coexpression cluster analysis across developmental stages

In addition to GCN analysis, differential gene expression analysis was performed using the default Wilcox test provided in Seurat to gauge the magnitude and specificity of genes towards cell clusters based on their expression (Supplementary Table 3 and Supplementary Fig. 3). This coexpression analysis identified several gene clusters which had stage-specific expression profiles including 1, 4, 6, 11, 12, 15, 17, 18, 19, 21, 22, 23, 25, 26, 29, and 30, which were associated with neurons, the fat body, malphigian tubules, the midgut, and cuticle (Table 4). More specifically, 5 out of 9 clusters related to neurons (C9, C14, C18, C19, and C49) were found at both stages. Two clusters associated with hemocytes (C20 and C43) were also found at both stages that might be associated with phagocytic cells involved in ingesting cell debris during metamorphosis. Sixteen clusters related to cuticle were identified in pupa at day 15 (C15, C21, C22 C31, C38, C41, C42, C45, C46, C47, C52, C55, C56, C58, C59, and C62) compared to four clusters at prepupa at day 11 (C36, C38, C39, and C51), this probably reflects the relative simplicity of the prepupal cuticle in contrast to the different types of cuticle required in the adult insect with it's differences in thickness, architecture, flexibility, and incorporation of sensory receptors. Six cell clusters were found in prepupa at day 11 (C1, C3, C4, C10, C13, and C25) in comparison to a single cluster (C2) in pupa at day15; this may reflect increased storage of nutrients in the larva or differences in synthesis of hemolymph proteins or detoxification.

## Discussion

Two strategies have previously been adopted by other researchers studying development using scRNA-Seq. The first involves scRNA-Seq of whole organisms and the second of focusing on individual tissues. Here, we adopt the former approach which has proven useful in the exploration of cell types of model organisms of a similar scale and biological complexity, such as Cnidaria (Sebé-Pedrós, Saudemont, et al. 2018), *C. elegans* (Packer et al. 2019), and zebrafish (Farnsworth et al. 2020), where the cell diversity is largely unknown. In this study, we constructed a single-cell atlas spanning two developmental stages of the worker honey bee (prepupa at day 11 and pupa at day 15). The cell types and gene expression signatures we have detected reflect the major tissue rearrangements that occur during metamorphosis. Honey bees are holometabolous and much of the knowledge for pupal development in holometabolous insects is based on the model organism *D. melanogaster* (Thompson 2021). During metamorphosis, tissues can degenerate if they are not present in the adult (e.g. head gland), be remodeled without complete cell replacement (e.g. fat body) or generate a new adult structure (e.g. antenna, eyes, legs, and wings develop from undifferentiated cells in imaginal discs) (Tettamanti and Casartelli 2019). Apart from the nervous system and the malpighian tubules, the cells that form most tissues of the larva are not used for the corresponding tissues of the adult and the imaginal organs of the adult develop from the imaginal discs. By choosing to analyze whole pupal stages with scRNA-Seq, we have facilitated the detection of gene expression profiles from the breakdown of larval tissues and the formation of adult tissues.

The cell-to-cell network grouped cells into 63 clusters across which cells from the two stages were differently distributed. Hence, clustering of cells revealed stage-specific cell types/subtypes i.e. certain cell types were entirely represented by cells from a single stage while other clusters comprised cells from both stages. The majority of cell clusters were entirely comprised of cells from S2, furthermore these cells had a greater number of genes expressed relative to cells from S1. These results suggest an expanding heterogeneity for the types of cells and genes, which define them and reflect the fact that most of the organs of the adult honey bee are present at S2 whilst at S1, a lower number of larval tissues are about to be degenerated, remodeled, or replaced. To study the genes that were associated with the cell clusters, we developed a novel approach to improve the biological signal representing intercell cluster variation. Briefly, this was done by averaging the reads across cells from the same cluster and applying filters on the expression values to address certain technical artifacts within the data including spikes in expression and the variation of lowly expressed genes. The approach enabled the construction of a GCN from scRNA-Seq data, which captures intercell type variation while minimizing intracell type and technical variations. The GCN comprised 32 clusters of coexpressing genes that were associated with a wide range of biology as determined using a combination of GO enrichment and literature mining to identify cell types and tissue-specific biology. Cell types and tissues identified were related to the brain, sensory organs, cuticle, muscle, fat body, blood, and alimentary canal. Gene coexpression signatures were identified that were not only unique to cell clusters but also those that were shared across clusters e.g. developmental stage and lineage-specific signatures. Some cell clusters would have proved impossible to identify based on using literature for *Apis* only due to the limitations of the available resources as such it was necessary to compare to *Drosophila* where most organs are evolutionary conserved and where a database for GO terms are present.

For this study, we developed a protocol that can be used to prepare single cells of honey bee worker pupae for scRNA-Seq. It was necessary to develop a new method because protocols used by previous studies were not successful in producing viable cells from honey bee pupae in our hands. For example, Davie et al. (2018) and Hung et al. (2020) used Dulbecco's phosphate-buffered saline for their single-cell suspensions while we found that using WH2 medium (Goblirsch et al. 2013), a medium for primary culture of honey bee cells, resulted in isolation of a viable single-cell population. It is likely that this method could be used to generate viable single cells from a variety of larvae e.g. Dipterans and other Hymenopterans and for other pupae that are not encased in a hard cocoon. The method will not be suitable for insects with a fully formed chitin exoskeleton e.g. adult honey bees and the use might be further limited by the suitability of WH2 medium for the cells of larvae and pupae of other species. In addition, many honey bee tissues were either not detected or not identified in our analysis e.g. endocrine system, salivary glands, hypopharyngeal glands, esophagus, honey sac, small intestine, heart, rectum, sting, and ovary. This might be because there is insufficient scientific literature relevant to these pupal stages for identification (7 of the 63 clusters remain unidentified) or it might be that the protocol was not successful in obtaining particular cell types. It is surprising that there are noticeably few cells from the ventriculus (midgut) despite the relatively large size of this organ in the adult bee, and it therefore seems likely that a more vigorous homogenization or longer digestion might yield more cells from the midgut. Unfortunately, there are currently no comparable protocols and datasets for the honey bee with which to compare our results. As such, we can only judge the success of the protocol based on our own observations and analysis of the data. Further research could address a wider developmental series and ascertain the efficacy of the protocol as the cuticle toughens in the later pupal stages.

With the lack of a gene expression atlas for the honey bee, this study provides an initial step in determining the cellular heterogeneity, which can only be improved upon by sequencing more samples/cells, cross-species comparisons and analysis of gene expression experiments. This study will be of benefit to the construction of more comprehensive gene expression atlases by demonstrating that pupae can be analyzed at the single-cell level, which can be potentially extended to larvae and dissected adult organs e.g. brain. Furthermore, the dataset could be used in conducting cross-species comparisons for development, as has been done for Cnidaria (Sebé-Pedrós, Saudemont, et al. 2018), to study the evolution of certain cell types.

## Conclusions

In summary, we have demonstrated that a gene expression atlas of the whole honey bee at the level of single cells is possible at prepupal and pupal stages. We have developed approaches from single-cell isolation to the analysis of the resultant scRNA-Seq data using GCN. Through this process, we have identified several potential cell types and their associated gene signatures which are supported by enrichment analysis, and previous experimental evidence from the literature or databases. The gene lists associated with the cell clusters will be of benefit to future analyses, particularly for transcriptomic studies in whole pupae and for functional annotation of the honey bee genome.

## Supplementary Material

jkad178_Supplementary_Data

## Data Availability

The dataset described in this manuscript has been deposited in the European Nucleotide Archive (Project: PRJEB45881) and in ArrayExpress (BioStudy: E-MTAB-13158). Supplemental material available at G3 online.
