# Supplementary figures and images for: Cellular heterogeneity of the developing worker honey bee (Apis mellifera) pupa: a single cell transcriptomics analysis
Source: G3 (Bethesda). 2023 Aug 7;13(10):jkad178. doi: 10.1093/g3journal/jkad178 (PMC10542211; doi:10.1093/g3journal/jkad178)

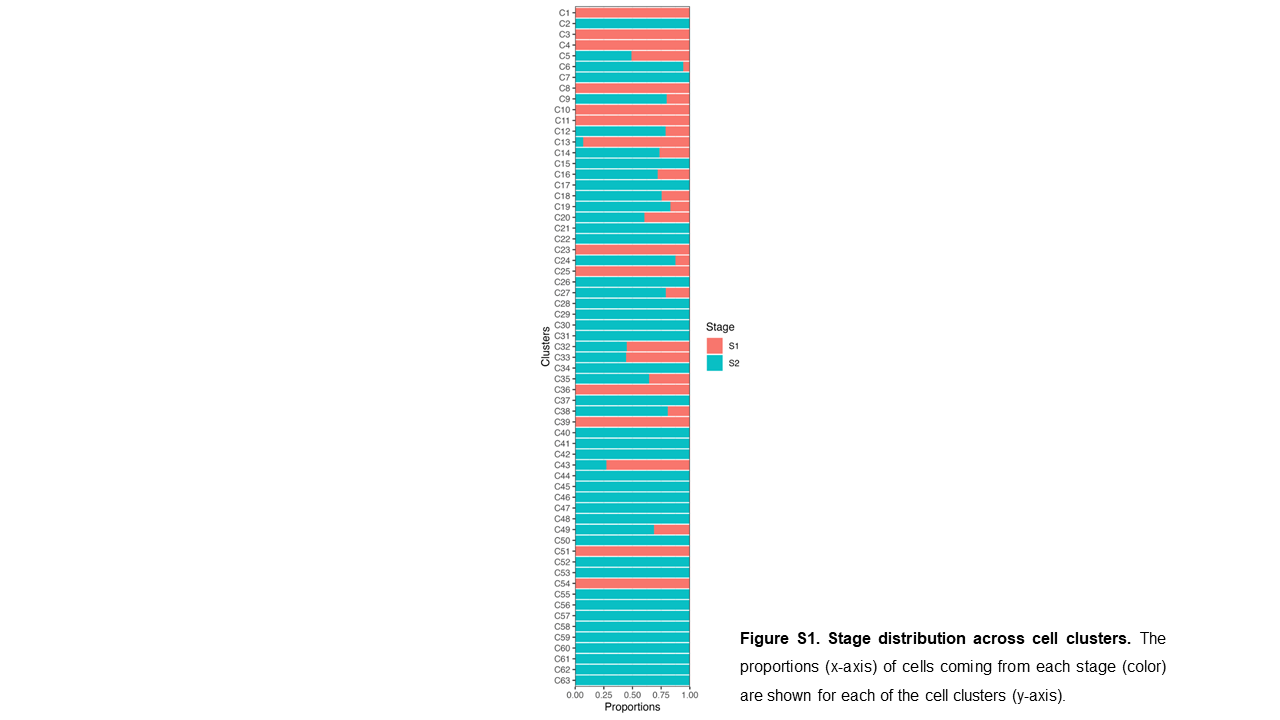

Supplement: jkad178_Supplementary_Data [file jkad178_supplementary_data.zip › Figure_S1_G3-2023-404389.png]

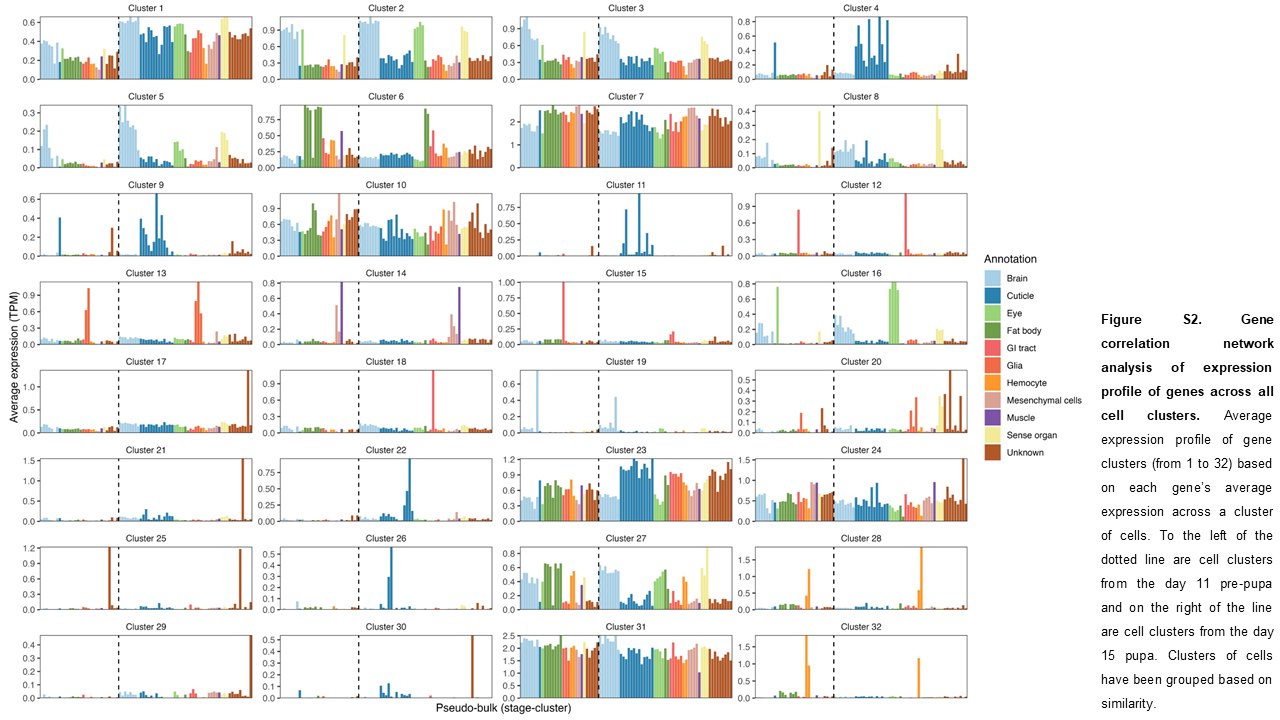

Supplement: jkad178_Supplementary_Data [file jkad178_supplementary_data.zip › Figure_S2_G3-2023-404389.png]

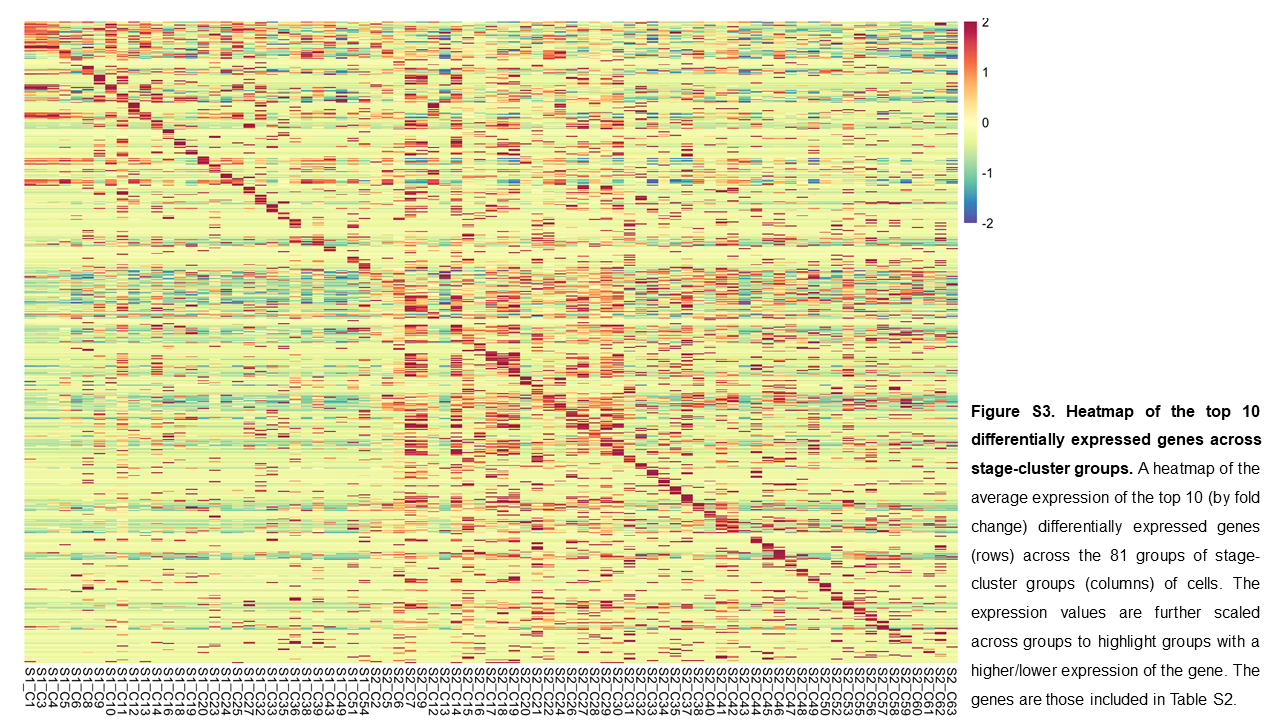

Supplement: jkad178_Supplementary_Data [file jkad178_supplementary_data.zip › Figure_S3_G3-2023-404389.png]
